# Antineutrophil cytoplasmic antibody-positive pauci-immune glomerulonephritis associated with mantle cell lymphoma 

**DOI:** 10.5414/CNCS109036

**Published:** 2017-02-03

**Authors:** Kana N. Miyata, Nazia A. Siddiqi, Lawrence P. Kiss, Nikolas B. Harbord, James F. Winchester

**Affiliations:** 1 Department of Internal Medicine, Division of Nephrology and Hypertension,; 2 Department of Pathology, Mount Sinai Beth Israel, New York, NY, and; 3 Department of Internal Medicine, Division of Nephrology and Hypertension, Harbor UCLA Medical Center, Torrance, CA, USA

**Keywords:** acute kidney injury, mantle cell lymphoma, crescent, lymphomatous infiltration of the kidney

## Abstract

Renal involvement in non-Hodgkin lymphoma, especially mantle cell lymphoma (MCL) is rare. A 77-year-old man presented with acute kidney injury (AKI), which rapidly progressed to dialysis dependence. Kidney biopsy revealed patchy B-cell lymphocytic aggregates in the interstitium, which were positive for cyclin D1, consistent with atypical CD5-negative MCL as confirmed by the detection of translocation t(11;14) by FISH. Crescents were noted in 3 of 26 glomeruli; while PR-3 antineutrophil cytoplasmic antibody (ANCA) positivity and negative immunofluorescence suggested an additional pauci-immune (rapidly progressive) glomerulonephritis pattern of injury. Patient received chemotherapy (cyclophosphamide, vincristine, and prednisone), which improved his renal function and allowed for discontinuation of hemodialysis. However, he died from pulmonary hemorrhage 8 months after initial presentation. This is the first reported case of a patient with coexistence of renal MCL infiltration and ANCA-positive pauci-immune glomerulonephritis.

## Introduction 

Mantle cell lymphoma (MCL) is predominantly a disease of elderly men and is characterized by its aggressive form of non-Hodgkin lymphoma (NHL) with short median survival of 3 – 4 years [1]. 

Lymphoma can involve the kidneys in various ways. Acute kidney injury (AKI) related to lymphoma can be from direct obstruction of the ureters or renal artery, renal vein thrombosis, lymphomatous infiltration of the kidneys, or paraneoplastic glomerulonephritis. It can also be from the indirect effect of hypercalcemia, bone invasion, paraproteinemia, and amyloid, or from treatment such as radiation nephritis and uric acid nephropathy [2, 25]. 

We present a case of AKI with biopsy-proven concomitant MCL infiltration to the kidneys and paraneoplastic antineutrophil cytoplasmic antibody (ANCA)-positive pauci-immune glomerulonephritis with crescent formation. 

## Case 

A 77-year-old Filipino man presented with worsening kidney function. His medical history was significant for chronic kidney disease, hypertension, hypothyroidism, and bladder cancer (low-grade urothelial tumor) for which he underwent transurethral resections 3 times until 4 months prior to this admission. He had a 10-pack-year smoking history but denied any alcohol or drug use. Two months prior, he was noted to have a 2.2 × 1.7 cm right lung mass on chest X-ray, and a CT scan showed diffuse lymphadenopathy in the neck, chest, abdomen, and pelvis. The result of bronchoscopy with biopsy was inconclusive. 

He was found to have acute kidney injury (AKI) (serum creatinine of 4.5 mg/dL from 2.2 mg/dL 1 week prior) by his primary care physician and was sent to our medical center. 

On admission, he complained of fatigue and decreased appetite for several months. His vital signs showed temperature of 36.7 °C, blood pressure of 146/73 mmHg, pulse rate of 83 beats/min, and oxygen saturation of 98% on room air. He was a small thin old man in no acute distress. Physical examination revealed II/VI holosystolic murmur, mild crackles at left lower lung base, palpable nontender submandibular and right axillary lymph nodes, no peripheral edema, and no skin rash. Urinalysis showed protein 1+, blood 2+, WBC 3/HPF, RBC 73/HPF with many dysmorphic RBCs, positive eosinophils, and granular casts. Urine protein/creatinine ratio was 2.9 g/gCr. Laboratory studies showed serum hemoglobin of 8.3 g/dL, urea nitrogen of 43 mg/dL, and creatinine of 5.3 mg/dL. HIV and hepatitis panel were negative. He had low C3 49 mg/dL and C4 16 mg/dL, positive ANA 1 : 160, negative MPO-ANCA, and positive PR-3 ANCA (5.5 AU/mL). Serum and urine protein electrophoresis were unremarkable. Kidney ultrasound showed slightly enlarged kidneys for his height (right 12.4 cm and left 11.4 cm) with increased echogenicity. Hospital course was complicated by pulmonary edema associated with a non-ST-elevation myocardial infarction. Kidney function continued to decline requiring hemodialysis on hospital day 16. 

**Figure 1.**
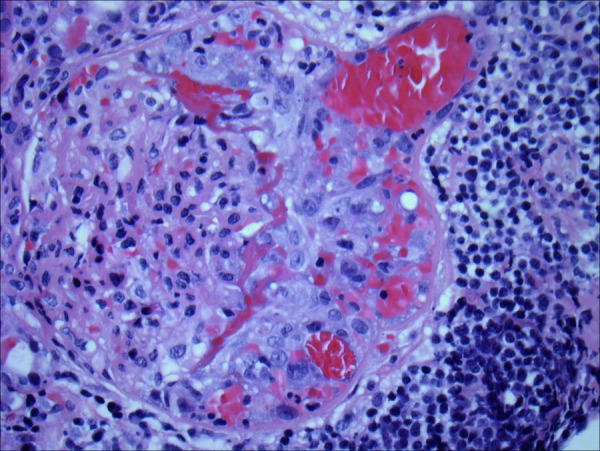
Kidney biopsy showing a glomerulus with cellular crescent formation (H & E stain; 400×).

**Figure 2.**
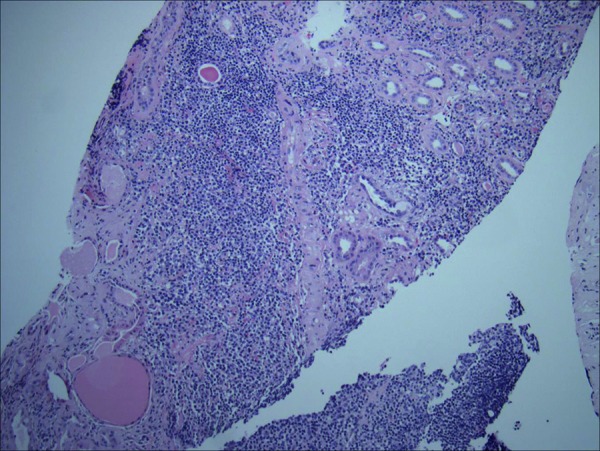
Kidney biopsy showing interstitial infiltrate of atypical lymphocytes (H & E stain; 100×).

**Figure 3.**
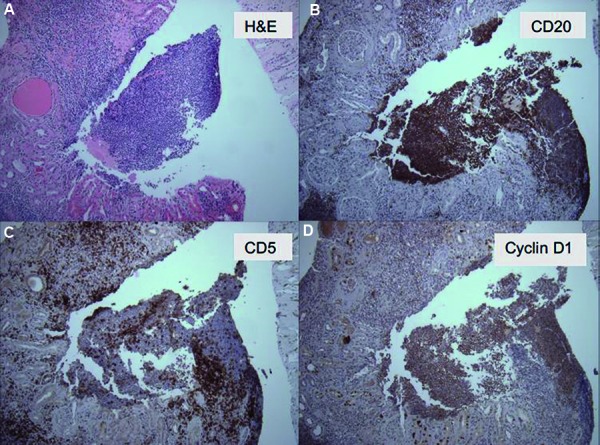
Kidney biopsy showing atypical lymphocytes in interstitium (A: H & E stain; 100×). Immunohistochemistry markers were positive for CD20 (B: 100×) and cyclin D1 (D: 100×), and negative for CD5 (C: 100×).

The kidney biopsy contained 26 glomeruli, of which 2 were globally sclerosed. Three glomeruli showed cellular crescent formation with epithelial cells and admixed inflammatory cells with scanty fibrin (Figure 1). The remaining glomeruli were roughly normal in size with normal cellularity. The mesangial areas had normal amounts of matrix and cellularity. There was no evidence of endocapillary proliferation, glomerulitis, or double contour formation. There was moderate tubular atrophy and interstitial fibrosis occupying ~ 20 – 40% of the cortical area. There were patchy dense monotonous lymphocytic aggregates and many separate areas with mixed inflammation including frequent plasma cells, occasional eosinophils, and no neutrophils (Figure 2). The lymphoid aggregates consisted of atypical mature lymphoid cells with irregular nuclear contours that were predominantly B-cells, positive for CD20 and cyclin D1, negative for CD3, CD5, and CD 10 (Figure 3). The findings supported a diagnosis of atypical CD5-negative mantle cell lymphoma confirmed by the detection of translocation t(11;14) by FISH (fluorescence in situ hybridization). Immunofluorescence and electron microscopy did not show immune complex deposits. 

Subsequent bone marrow biopsy did not show bone marrow involvement by lymphoma. The patient was diagnosed with stage 4EB mantle cell lymphoma (diffuse lymphadenopathy, splenic and kidney involvement, lung lesions) with coexistence of renal infiltration by MCL and pauci-immune glomerulonephritis. 

Given his age and multiple comorbidities, the patient received 6 cycles of palliative chemotherapy (IV cyclophosphamide 325 mg/m^2^ on day 1, IV vincristine 1.4 mg/m^2^ on day 1, and oral prednisone 100 mg daily on days 1 – 5; every 3 weeks). After the completion of chemotherapy, he was noted to have regained some renal function, and hemodialysis was discontinued. However, he died from pulmonary hemorrhage at 8 months from the initial presentation. 

## Discussion 

We present a rare case of AKI caused by MCL rapidly leading to end-stage renal disease. An interesting point of this case is the coexistence of the two possible causes of AKI; lymphoma infiltration into the interstitium and PR3-ANCA-positive pauci-immune glomerulonephritis likely as a paraneoplastic manifestation. 

The differential diagnosis of AKI related to malignant lymphoma is broad. Direct obstruction of the ureters, renal arteries, and veins by tumor masses can be diagnosed by imaging tests, and treatment-related AKI is usually obvious from the treatment history. The enlargement of kidneys bilaterally may be seen with the direct lymphomatous infiltration as in this case. However, kidney biopsy is usually helpful for the accurate diagnosis of the lymphoma subtypes and the clarification of the extent and location of the infiltration, which may influence the prognosis [3]. Paraneoplastic glomerulonephritis typically requires kidney biopsy for the diagnosis. Da’as et al. [8] reported that 83 patients out of 700 patients with NHL or chronic lymphocytic leukemia (CLL) had manifestations of renal failure. The overall incidence of kidney involvement in MCL is not known, likely because MCL is a rare disease, occurring only in 3 – 7% of NHLs in United States and Europe [26]. 

In a previously published large case series, Richmond et al. [4] identified lymphoma cells’ renal parenchymal infiltration in 34%of all the lymphoma autopsy cases, but clinically significant renal failure was observed in less than 10% of the patients with renal lymphoma infiltrate. Although rare, AKI, leading to ESRD, can be caused solely by lymphoma infiltration as reported by Lee et al. [5]. 

To our knowledge, this is the first report of biopsy-proven ANCA-positive pauci-immune glomerulonephritis with crescent formation associated with MCL. To date, there are 21 reported cases of MCL with renal involvement; 10 cases with renal MCL infiltration, 3 cases with proliferative glomerulonephritis, 4 cases with membranoproliferative glomerulonephritis, 2 cases with minimal change disease, 2 cases with focal segmental glomerulosclerosis, 1 case with immune complex-mediated glomerulonephritis, and 5 cases with ANCA-negative crescent formation (Table 1). Out of 5 cases with crescents, 3 cases had concomitant lymphomatous infiltration of tubulointerstitium as seen in our case [15, 20, 21]. Out of the 21 cases reported, 15 cases mention the result of ANCA and all were reported negative. 

It is known that the risk of malignancies is increased in patients with ANCA-associated vasculitis compared to the general population [27]. The association of solid tumor malignancies, such as kidney, lung, or colon cancer, and paraneoplastic ANCA-associated vasculitis has been published sporadically in case reports [28]. Li et al. [17] identified 20 NHL patients with renal involvement, among which 2 patients had positive PR3-ANCA (1 patient with T/NK cell lymphoma and another with chronic lymphocytic leukemia/small lymphocytic lymphoma). In those studies, ANCA-associated vasculitis occurred concurrently or preceded the cancer diagnosis. Pathophysiological mechanism of paraneoplastic glomerulonephritis remains largely undetermined. Hypotheses include dysregulation of T-cell immunology, vascular endothelial growth factor (VEGF) and VEGF-receptor dysregulation, increased cytokine levels, antibody production by the neoplasm, and deposits of malignancy-related antigens [20, 28, 29]. 

It is important to note that the current standard treatment option for ANCA-associated vasculitis is a part of the chemotherapy regimen for MCL. Cyclophosphamide or rituximab in addition to corticosteroids are usually used for both diseases, though the dosing may be different. Renal recovery in our case might be attributed to the resolution of both of the histologic findings by his chemotherapy regimen. Interestingly, most of the case reports of MCL-related kidney disease have good renal outcomes after the treatment of MCL, though it tends to recur when MCL recurs (Table 1). This is another clue that shows renal injury is a paraneoplastic feature and not de-novo kidney disease. 

Learning points of this case are (1) lymphoma infiltration and/or glomerular disease associated with lymphoma should be suspected as a differential diagnosis for AKI with an underlying hematologic disease, and (2) early detection by kidney biopsy and initiation of cancer treatment can possibly change the patients’ renal and/or overall outcomes. 

## Conflict of interest 

Authors declare no conflict of interest. 

**Table 1.** Renal manifestations of mantle cell lymphoma in previous case reports.

Case	Age (years)	Gender	Lymphomatous infiltration to tubulointerstitium	Glomerular findings	ANCA	Treatments	Renal improvement after MCL treatments	Reference
1	72	F	Yes	No	Unknown	Prednisone, vincristine, prednimustine, mitoxantrone	Yes; discontinuation of HD	Baldus et al. 1996 [6]
2	77	M	No	Crescent formation (1 out of 8 glomeruli)	Neg	CHOP	Yes; discontinuation of HD	Rerolle et al. 1999 [7]
3	52	M	No	Proliferative glomerulonephritis	Unknown	IV methylprednisolone, adriamycin, cyclophosphamide, and prednisone	Yes; discontinuation of HD, kidney function returned to normal	Da’as et al. 2001 [8]
4	69	M	Yes (AIN with predominant B lymphocyte infiltration)	No	Neg	Prednisolone	Yes; S-Cr improved to 1.0 mg/dL	Wu et al. 2002 [9]
5	75	M	No	Proliferative glomerulonephritis with crescents (3 out of 8 glomeruli)	Neg	IV methylprednisolone, oral cyclophosphamide, prednisolone, and azathioprine	Yes; discontinuation of HD, S-Cr improved to 400 µmol/L (4.5 mg/dL)	Karim et al. 2004 [10]
6	68	M	No	Endocapillary proliferative glomerulonephritis	Neg	Oral prednisolone and chlorambucil	Yes; discontinuation of HD, S-Cr improved to 220 µmol/L (2.5 mg/dL)	Karim et al. 2004 [10]
7	80	M	Yes	MPGN, cryoglobulinemia	Neg	Rituximab, prednisolone	Unknown**	Hill et al. 2004 [11]
8	73	M	Yes	No	Unknown	Unknown	Unknown	Colak et al. 2004 [12]
9	68	M	No	FSGS	Neg	IV cyclophosphamide, plasma exchange, R-CVP	Yes; discontinuation of HD, S-Cr improved to 124 µmol/L (1.4 mg/dL).	Wong et al. 2007 [13]
10	76	M	Yes	No	Neg	IV methylprednisolone and oral thalidomide	Yes; S-Cr decreased to 269 µmol/L (3.0 mg/dL) but deteriorated again.	Davies et al. 2007 [14]
11	69	M	Yes	No	Neg	None	N/A*	Lee et al. 2012 [5]
12	59	M	Yes	MPGN with crescents (2 out of 10 glomeruli)	Neg	IV methylprednisolone, oral prednisone, IV cyclophosphamide	Yes; discontinuation of HD, S-Cr improved to 79.56 µmol/L (0.9 mg/dL)	Lubas et al. 2013 [15]
13	68	M	No	MPGN	Neg	Rituximab, cyclophosphamide, vincristine, doxorubicin, and dexamethasone	Yes; discontinuation of HD, S-Cr improved to 0.5 mg/dL	Chu et al. 2013 [16]
14	65	M	No	MPGN	Neg	CHOP	Yes; S-Cr improved to 101 µmol/L (1.1 mg/dL)	Li et al. 2014 [17]
15	55	F	No	MCD	Neg	CHOP, methotrexate, HSCT	Yes	Khow et al. 2014 [18]
16	56	M	No	MCD	Unknown	R-COP	Yes; in remission	Kofman et al. 2014 [19]
17	46	F	Yes	Crescent formation (most of the glomeruli)	Neg	COP	Yes; S-Cr improved to 113.6 µmol/L (1.3 mg/dL)	Wang et al. 2014 [20]
18	54	M	Yes	Crescent formation (2 out of 5 glomeruli)	Neg	CHOP	Yes; S-Cr improved to < 3 mg/dL	Peddi et al. 2015 [21]
19	77	M	Yes	MPGN	Unknown	Rituximab, prednisone	Yes; S-Cr improved to 2.5 mg/dL	Sekulic et al. 2015 [22]
20	58	M	No	Immune complex glomerulonephritis	Neg	R-CHOP	Yes; S-Cr improved to 1.0 mg/dL	Abeysekera et al. 2015 [23]
21	67	M	No	FSGS	Unknown	Prednisolone, cyclosporine	Yes; S-Cr improved to 108 µmol/L (1.22 mg/dL) but disease relapsed	Hindocha et al. 2015 [24]
22	77	M	Yes	Crescent formation (3 out of 26 glomeruli)	Yes	COP	Yes; discontinuation of HD	Miyata et al. 2016 (present case)

*N/A = Patient refused chemotherapy and continued hemodialysis. **Unknown = systemic chemotherapy was not given considering the patient’s age and fragility. ANCA = antineutrophil cytoplasmic antibody; MCL = mantle cell lymphoma; HD = hemodialysis; Neg = negative; S-Cr = serum creatinine; FSGS = focal and segmental glomerular sclerosis; MPGN = membranoproliferative glomerulonephritis; MCD = minimal change disease; AIN = acute interstitial nephritis; IV = intravenous; CHOP = cyclophosphamide, doxorubicin, vincristine, prednisone; R-CVP = rituximab, cyclophosphamide, vincristine, and prednisolone; HSCT = autologous hematopoietic stem cell transplantation; R-COP = rituximab, cyclophosphamide, vincristine, and prednisone; COP = cyclophosphamide, vincristine, and prednisone.
